# Supplementary figures and images for: A recombinant protein containing influenza viral conserved epitopes and superantigen induces broad-spectrum protection
Source: eLife. 2021 Nov 16;10:e71725. doi: 10.7554/eLife.71725 (PMC8635977; doi:10.7554/eLife.71725)

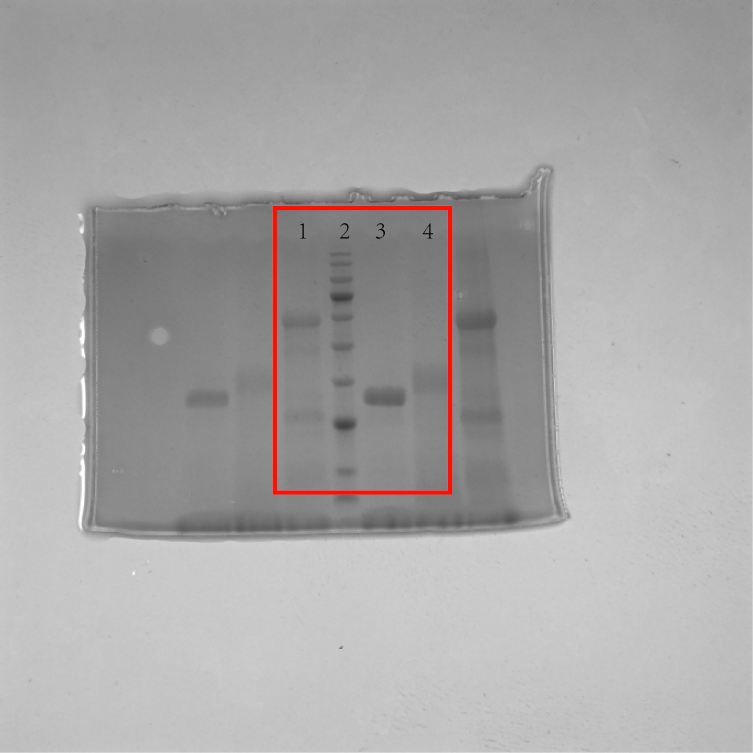

Supplement: Figure 1—source data 1. — This folder contains the original files of the full raw unedited gel (named original gel) and the relevant bands clearly labeled gel (named labeled gel). The information can be found in Figure 1 legend, as well as in Methods. [file elife-71725-fig1-data1.zip › labelled gel .tif]

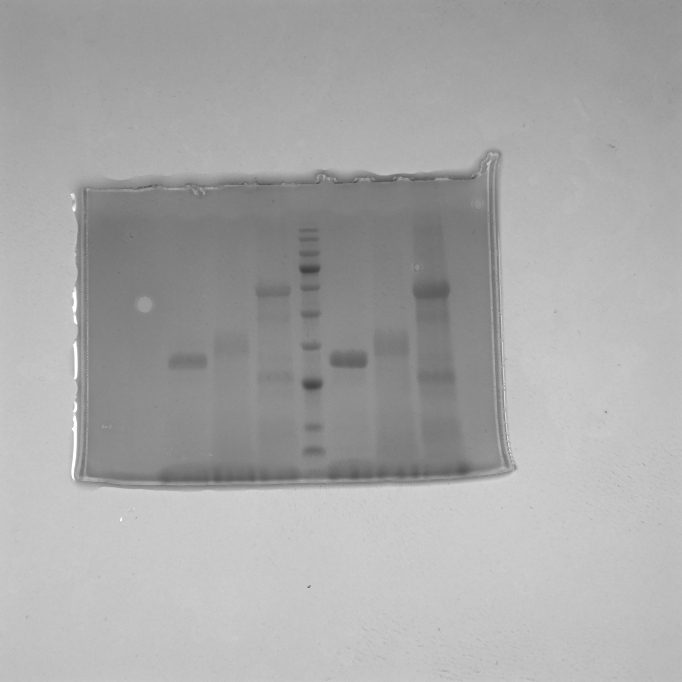

Supplement: Figure 1—source data 1. — This folder contains the original files of the full raw unedited gel (named original gel) and the relevant bands clearly labeled gel (named labeled gel). The information can be found in Figure 1 legend, as well as in Methods. [file elife-71725-fig1-data1.zip › Original gel .tif]

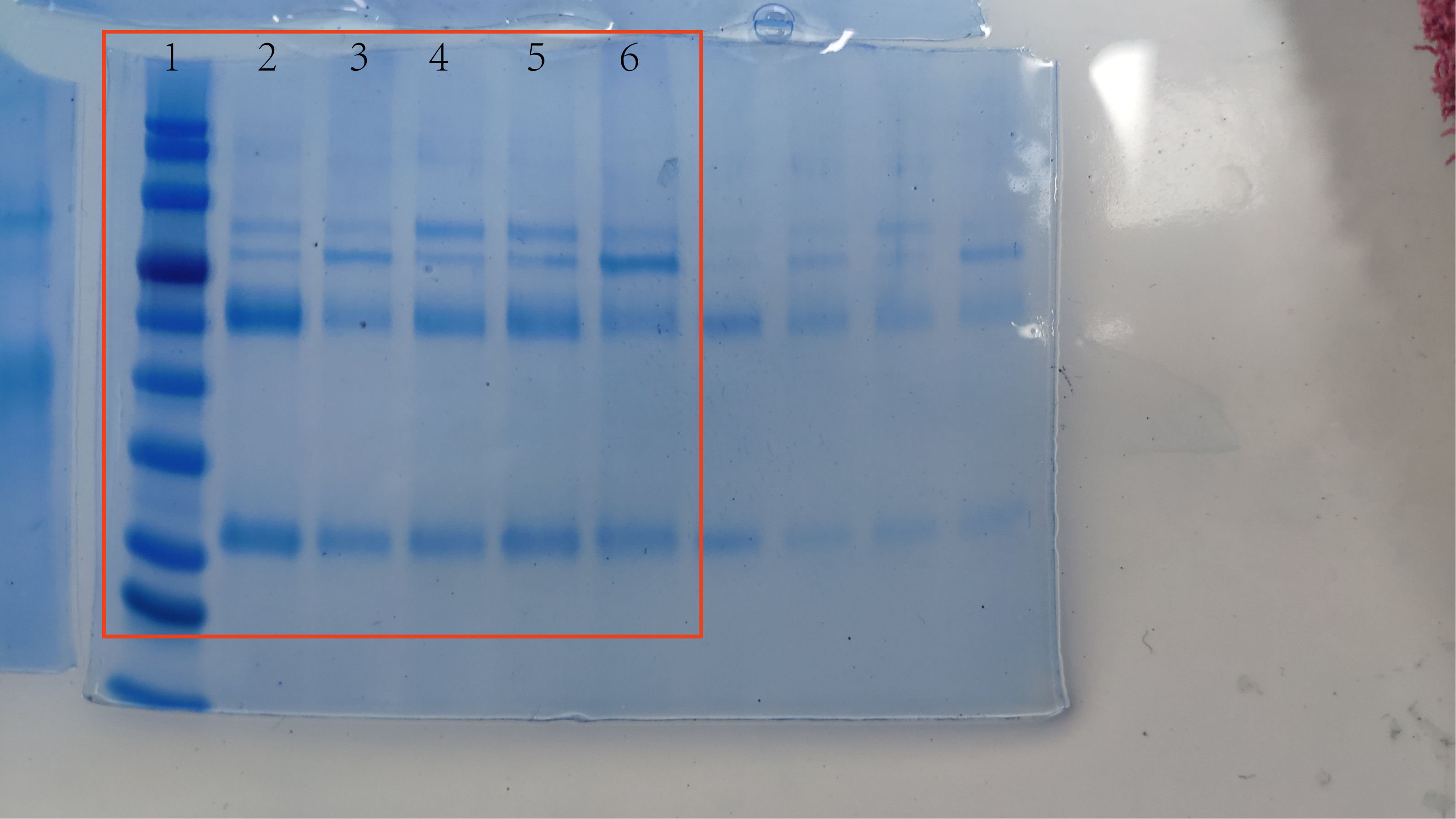

Supplement: Figure 6—figure supplement 1—source data 1. — This folder contains the original files of the full raw unedited gel (individual files are named ‘original gel (Line1-6)’, ‘original gel (Line7-15)’, ‘original gel (Line16-20)’), and the relevant bands clearly labeled gel (individual files are named ‘labelled gel (Line1-6)’, ‘labelled gel (Line7-15)’, ‘labelled gel (Line16-20)’). The information can be found in Figure 6—figure supplement 1 legends, as well as in Methods. [file elife-71725-fig6-figsupp1-data1.zip › Labelled gel(Line1-6).tif]

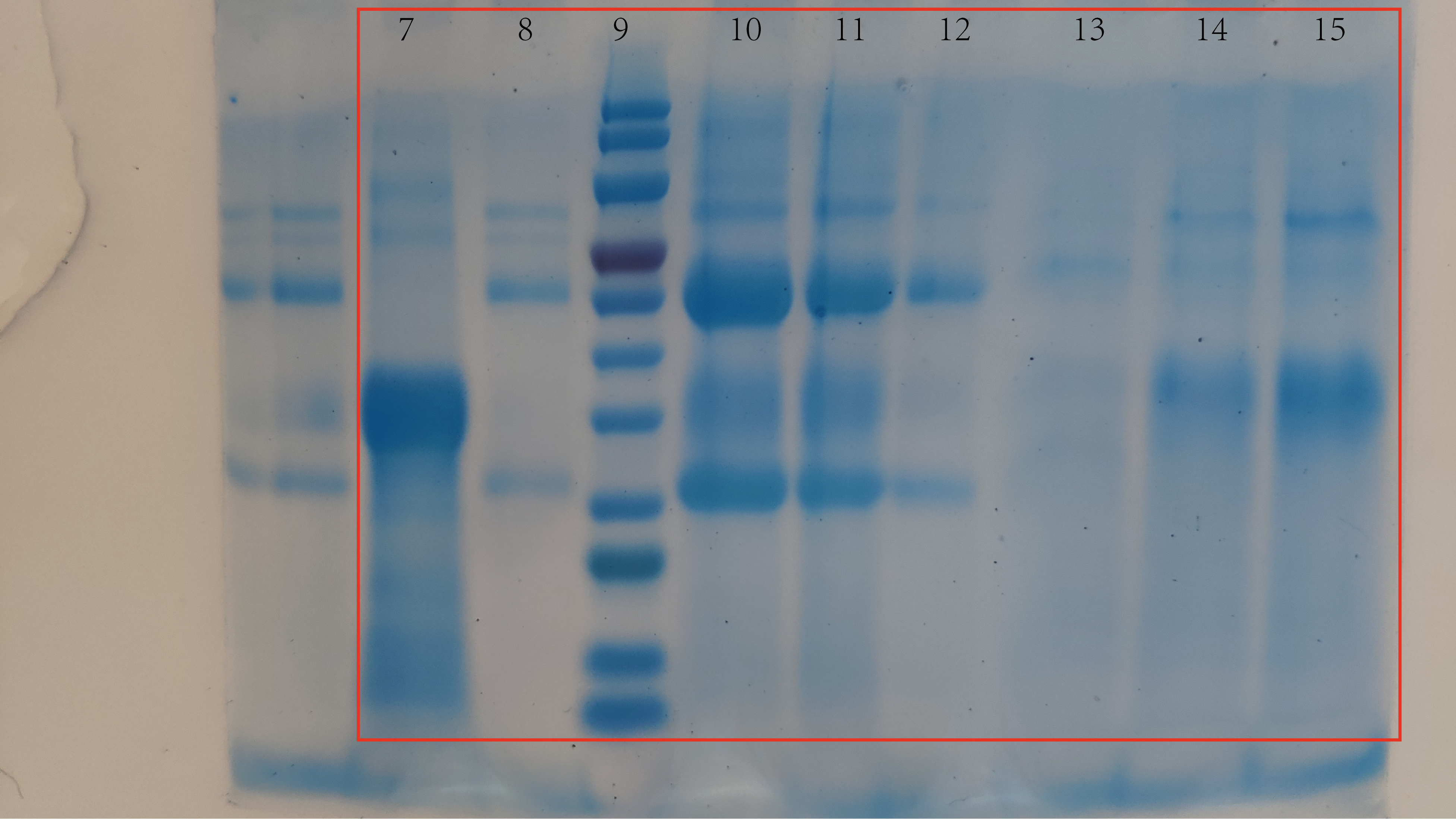

Supplement: Figure 6—figure supplement 1—source data 1. — This folder contains the original files of the full raw unedited gel (individual files are named ‘original gel (Line1-6)’, ‘original gel (Line7-15)’, ‘original gel (Line16-20)’), and the relevant bands clearly labeled gel (individual files are named ‘labelled gel (Line1-6)’, ‘labelled gel (Line7-15)’, ‘labelled gel (Line16-20)’). The information can be found in Figure 6—figure supplement 1 legends, as well as in Methods. [file elife-71725-fig6-figsupp1-data1.zip › Labelled gel(Line7-15).tif]

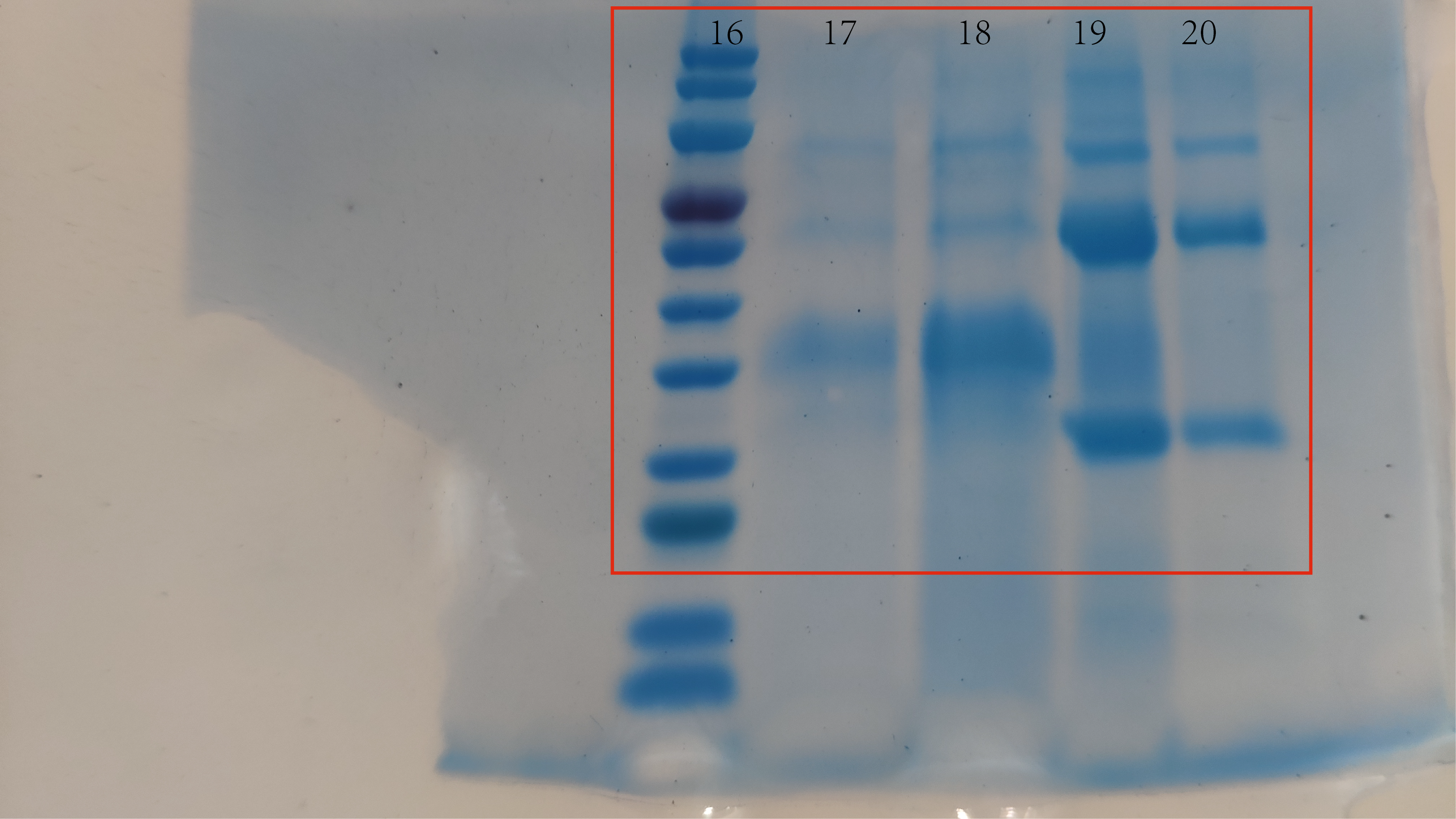

Supplement: Figure 6—figure supplement 1—source data 1. — This folder contains the original files of the full raw unedited gel (individual files are named ‘original gel (Line1-6)’, ‘original gel (Line7-15)’, ‘original gel (Line16-20)’), and the relevant bands clearly labeled gel (individual files are named ‘labelled gel (Line1-6)’, ‘labelled gel (Line7-15)’, ‘labelled gel (Line16-20)’). The information can be found in Figure 6—figure supplement 1 legends, as well as in Methods. [file elife-71725-fig6-figsupp1-data1.zip › Labelled gel(Line16-20).tif]

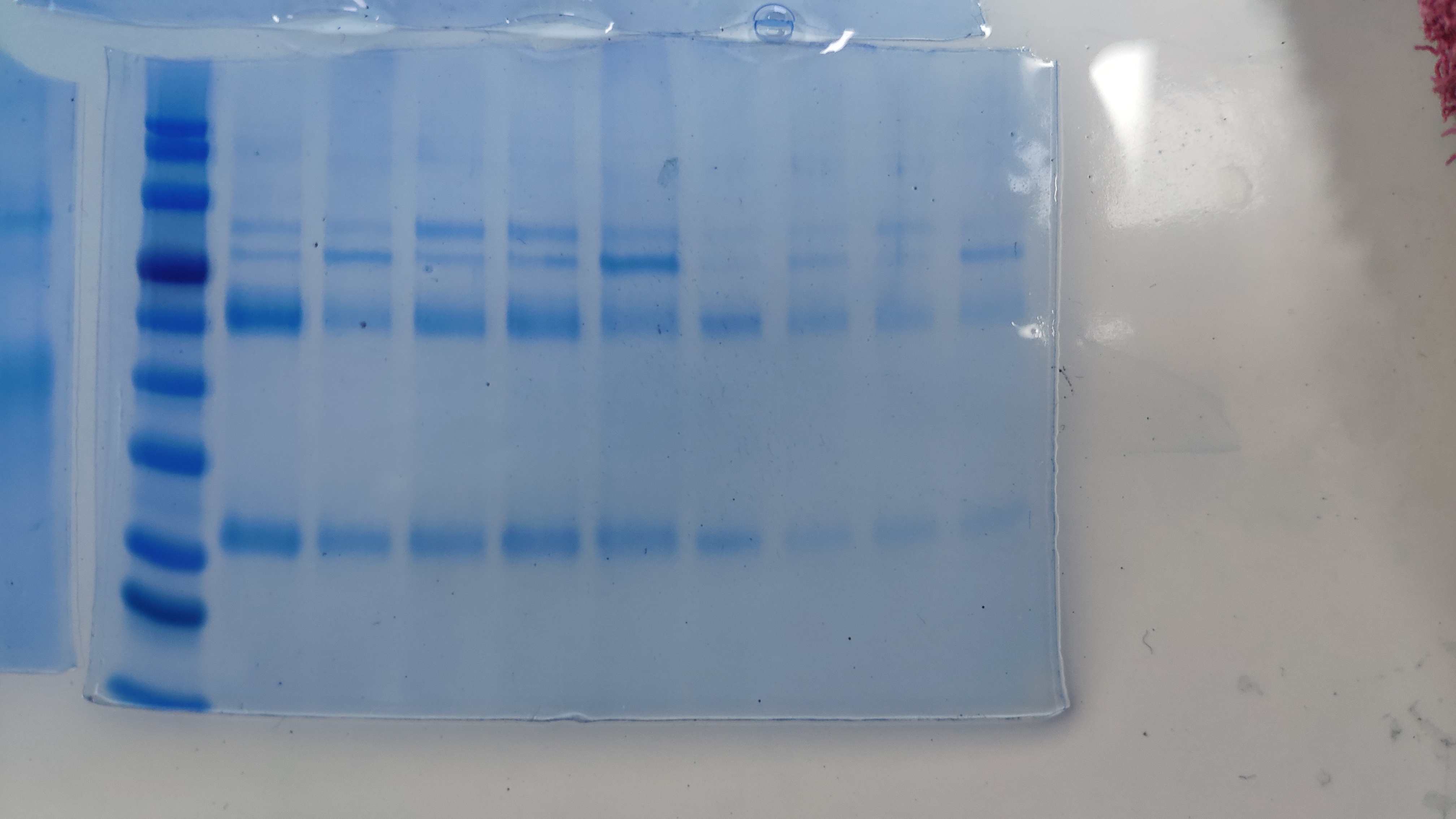

Supplement: Figure 6—figure supplement 1—source data 1. — This folder contains the original files of the full raw unedited gel (individual files are named ‘original gel (Line1-6)’, ‘original gel (Line7-15)’, ‘original gel (Line16-20)’), and the relevant bands clearly labeled gel (individual files are named ‘labelled gel (Line1-6)’, ‘labelled gel (Line7-15)’, ‘labelled gel (Line16-20)’). The information can be found in Figure 6—figure supplement 1 legends, as well as in Methods. [file elife-71725-fig6-figsupp1-data1.zip › Original gel(Line1-6).jpg]

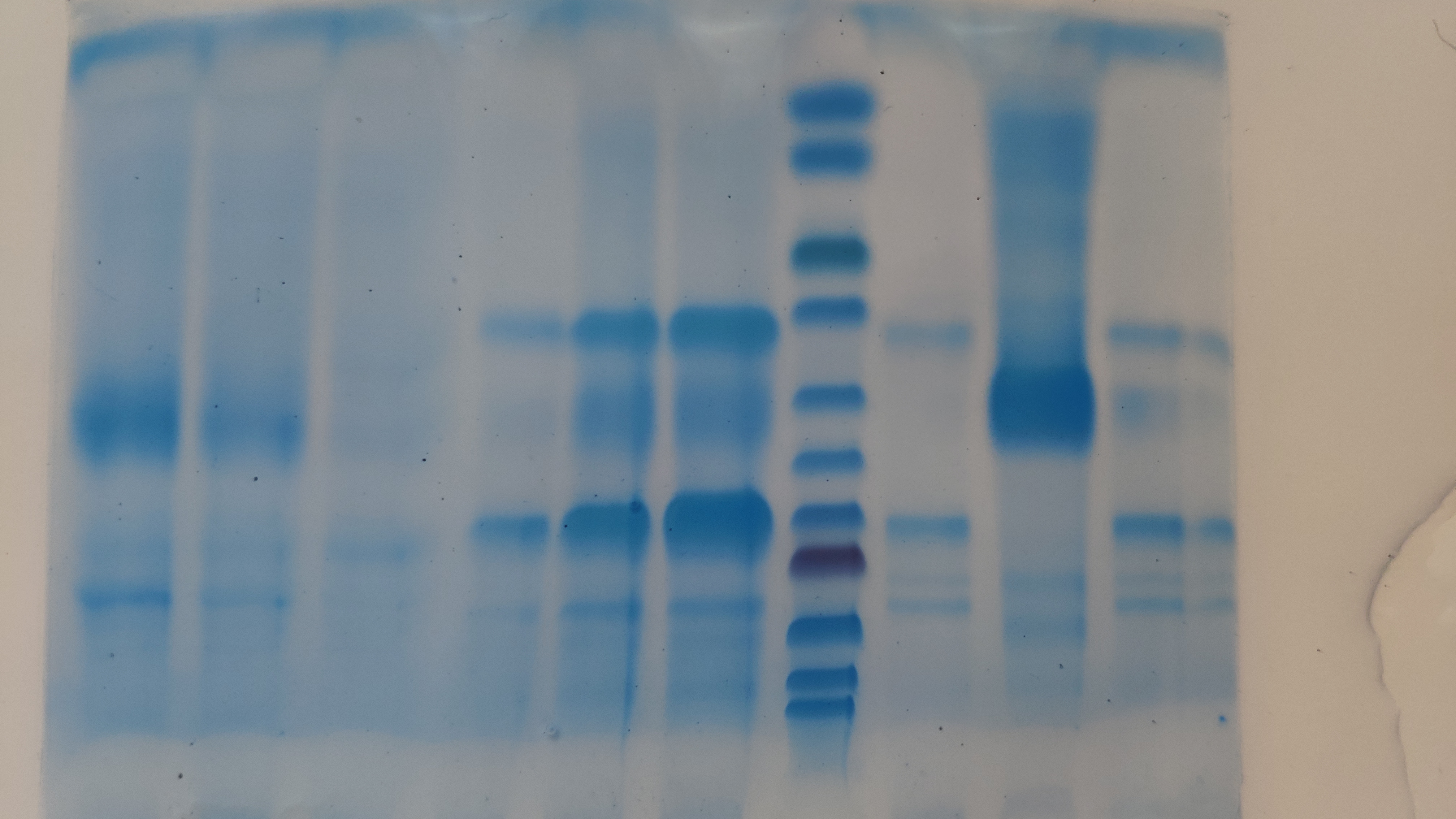

Supplement: Figure 6—figure supplement 1—source data 1. — This folder contains the original files of the full raw unedited gel (individual files are named ‘original gel (Line1-6)’, ‘original gel (Line7-15)’, ‘original gel (Line16-20)’), and the relevant bands clearly labeled gel (individual files are named ‘labelled gel (Line1-6)’, ‘labelled gel (Line7-15)’, ‘labelled gel (Line16-20)’). The information can be found in Figure 6—figure supplement 1 legends, as well as in Methods. [file elife-71725-fig6-figsupp1-data1.zip › Original gel(Line7-15).jpg]

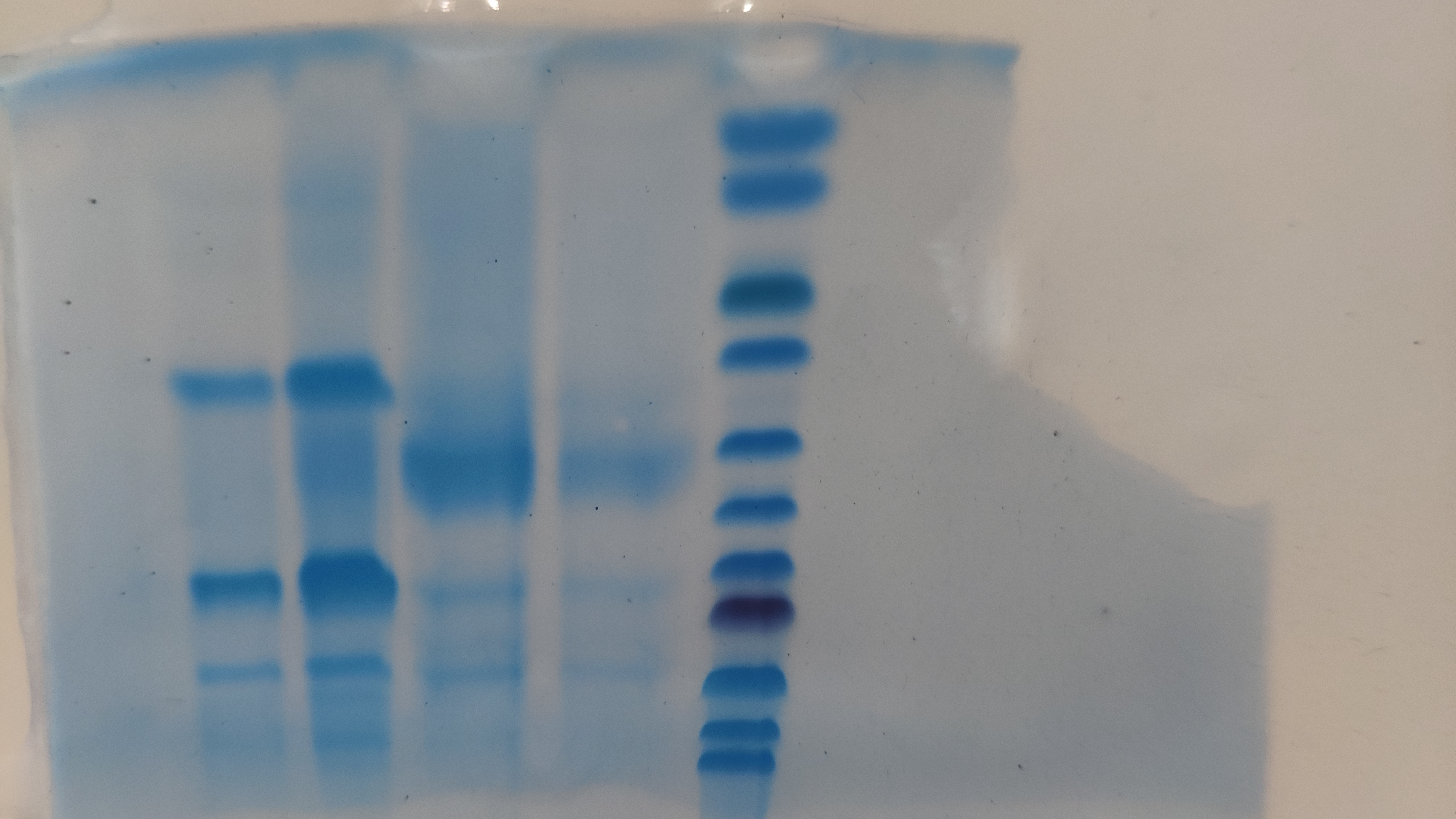

Supplement: Figure 6—figure supplement 1—source data 1. — This folder contains the original files of the full raw unedited gel (individual files are named ‘original gel (Line1-6)’, ‘original gel (Line7-15)’, ‘original gel (Line16-20)’), and the relevant bands clearly labeled gel (individual files are named ‘labelled gel (Line1-6)’, ‘labelled gel (Line7-15)’, ‘labelled gel (Line16-20)’). The information can be found in Figure 6—figure supplement 1 legends, as well as in Methods. [file elife-71725-fig6-figsupp1-data1.zip › Original gel(Line16-20).jpg]
