# Supplementary material for: A recombinant protein containing influenza viral conserved epitopes and superantigen induces broad-spectrum protection
Source: eLife. 2021 Nov 16;10:e71725. doi: 10.7554/eLife.71725 (PMC8635977; doi:10.7554/eLife.71725)
Supplement: Supplementary file 1. — (a) Neutralizing antibody titers detected by standard HI. Sera were collected from immunized mice on day 42 after immunization, and the neutralization assays were performed against MI/45(H1), HK/4801(H3), Influenza Virus Infectious NYMC BX-35 (Victoria), B/Phuket/3073/2013-like virus (Yamagata) influenza viruses. The hemagglutination status of each well was visually determined. The titers of each serum sample were defined as the reciprocal of the highest dilution where no hemagglutination was observed. (b) ELISA endpoint titers of HAs or split virion. On day 42 after immunization, breadth of the antibody response elicited by the recombinant protein NMHC was determined by ELISA of the pooled antisera against purified rHA proteins or split virion. Titer was defined as the highest dilution of serum antibodies at which the mean OD450 value of the experiment group was no less than 2.1 times of the control. [file elife-71725-supp1.docx]

**Supplementary Information for**

A recombinant protein containing influenza viral conserved epitopes and superantigen induces broad-spectrum protection

Yansheng Li^a,b,c^, Mingkai Xu^a,c,⁎^, Yongqiang Li^a,b,c^, Wu Gu^a,c^, Gulinare Halimu^a,b,c^, Yuqi Li^a,b,c^, Zhichun Zhang ^a,b,c^, Libao Zhou ^d^, Hui Liao ^d^, Songyuan Yao ^d^, Huiwen Zhang^a,c^, Chenggang Zhang^a,c^

^a^ Institute of Applied Ecology, Chinese Academy of Sciences, 72 WenHua Road, Shenyang 110016, PR China

^b^ University of Chinese Academy of Sciences, 19 YuQuan Road, Beijing 100049, PR China

^c^ Key Laboratory of Superantigen Research, Shenyang Bureau of Science and Technology, 72 WenHua Road, Shenyang 110016, PR China

^d^ Chengda Biotechnology Co.Ltd., Liaoning, China

Corresponding Author: *Mingkai Xu.

Email: mkxu@iae.ac.cn

**This PDF file includes:**

Supplementary File 1a and 1b

**Supplementary File 1a**. Neutralizing antibody titers detected by standard HI

| Immunization | | Serum dilution(1/X) | | | |
| --- | --- | --- | --- | --- | --- |
|  |  | H1N1 | H3N2 | Victoria | Yamagata |
| Day14 | NMHC | < 1:8 | < 1:8 | < 1:8 | < 1:8 |
|  | NMH | < 1:8 | < 1:8 | < 1:8 | < 1:8 |
|  | SEC2 | < 1:8 | < 1:8 | < 1:8 | < 1:8 |
|  | NMH+SEC2 | < 1:8 | < 1:8 | < 1:8 | < 1:8 |
|  | PBS | < 1:8 | < 1:8 | < 1:8 | < 1:8 |
|  | QIV | 1:128 | 1:16 | 1:64 | 1:32 |
|  |  |  |  |  |  |
| Day28 | NMHC | < 1:8 | < 1:8 | < 1:8 | < 1:8 |
|  | NMH | < 1:8 | < 1:8 | < 1:8 | < 1:8 |
|  | SEC2 | < 1:8 | < 1:8 | < 1:8 | < 1:8 |
|  | NMH+SEC2 | < 1:8 | < 1:8 | < 1:8 | < 1:8 |
|  | PBS | < 1:8 | < 1:8 | < 1:8 | < 1:8 |
|  | QIV | 1:256 | 1:256 | 1:256 | 1:256 |
|  |  |  |  |  |  |
| Day42 | NMHC | < 1:8 | < 1:8 | < 1:8 | < 1:8 |
|  | NMH | < 1:8 | < 1:8 | < 1:8 | < 1:8 |
|  | SEC2 | < 1:8 | < 1:8 | < 1:8 | < 1:8 |
|  | NMH+SEC2 | < 1:8 | < 1:8 | < 1:8 | < 1:8 |
|  | PBS | < 1:8 | < 1:8 | < 1:8 | < 1:8 |
|  | QIV | 1:256 | 1:25 | 1:512 | 1:256 |

Quadrivalent influenza vaccine (QIV) was regarded as positive control.

**Supplementary File 1b.** ELISA endpoint titers of HAs or split virion

| Vaccine | Mouse | Titer against | | | | | | | | | |
| --- | --- | --- | --- | --- | --- | --- | --- | --- | --- | --- | --- |
|  |  | H1N1 | | | H3N2 | | | H2N2 | H5N1 | H7N9 | H9N2 |
|  |  | IgG | IgG1 | IgG2a | IgG | IgG1 | IgG2a | IgG | IgG | IgG | IgG |
| NMHC | No.1 | 3200 | 1600 | 400 | 3200 | 1600 | 400 | 8192 | 8192 | 8192 | 8192 |
|  | No.2 | 6400 | 3200 | 400 | 3200 | 1600 | 200 | 8192 | 8192 | 8192 | 8192 |
|  | No.3 | 6400 | 3200 | 800 | 3200 | 800 | 200 | 4096 | 8192 | 8192 | 8192 |
|  |  |  |  |  |  |  |  |  |  |  |  |
| NMH | No.1 | 1600 | 1600 | 100 | 400 | 400 | 200 | 8192 | 2048 | 2048 | 4096 |
|  | No.2 | 1600 | 1600 | 100 | 400 | 400 | 200 | 8192 | 4096 | 2048 | 4096 |
|  | No.3 | 1600 | 1600 | 100 | 400 | 400 | 200 | 4096 | 4096 | 2048 | 8192 |
|  |  |  |  |  |  |  |  |  |  |  |  |
| SEC2 | No.1 | ＜10 | ＜10 | ＜10 | ＜10 | ＜10 | ＜10 | ＜10 | ＜10 | ＜10 | ＜10 |
|  | No.2 | ＜10 | ＜10 | ＜10 | ＜10 | ＜10 | ＜10 | ＜10 | ＜10 | ＜10 | ＜10 |
|  | No.3 | ＜10 | ＜10 | ＜10 | ＜10 | ＜10 | ＜10 | ＜10 | ＜10 | ＜10 | ＜10 |
|  |  |  |  |  |  |  |  |  |  |  |  |
| NMH  +SEC2 | No.1 | 6400 | 3200 | 100 | 1600 | 1600 | 100 | 8192 | 8192 | 4096 | 8192 |
|  | No.2 | 6400 | 3200 | 100 | 3200 | 3200 | 100 | 8192 | 8192 | 4096 | 8192 |
|  | No.3 | 6400 | 6400 | 100 | 3200 | 3200 | 200 | 8192 | 4096 | 4096 | 8192 |
|  |  |  |  |  |  |  |  |  |  |  |  |
| PBS | No.1 | ＜10 | ＜10 | ＜10 | ＜10 | ＜10 | ＜10 | ＜10 | ＜10 | ＜10 | ＜10 |
|  | No.2 | ＜10 | ＜10 | ＜10 | ＜10 | ＜10 | ＜10 | ＜10 | ＜10 | ＜10 | ＜10 |
|  | No.3 | ＜10 | ＜10 | ＜10 | ＜10 | ＜10 | ＜10 | ＜10 | ＜10 | ＜10 | ＜10 |
